# Breakfast consumption patterns and associated factors among adolescent high-school students in Tullo District, Eastern Ethiopia

**DOI:** 10.1371/journal.pone.0329608

**Published:** 2025-08-05

**Authors:** Natnael Teferi, Tara Wilfong, Dawit Firdisa, Samrawit Berihun, Behailu Hawulte

**Affiliations:** School of Public Health, College of Health and Medical Sciences, Haramaya University, Harar, Ethiopia; University of Tabuk, SAUDI ARABIA

## Abstract

**Background:**

There is growing proof to recommend eating breakfast has positive health and school-related outcomes for adolescents, including improved performance, attention, brain development, and physical growth. However, there is a dearth of evidence on the comprehensive understanding of breakfast consumption patterns and associated factors. Therefore, this study aimed to assess breakfast consumption patterns and their associated factors among adolescent high school students in the Tullo district, Eastern Ethiopia.

**Methods:**

An institution-based cross-sectional study design was conducted among 405 randomly selected adolescent high school students in the Tullo District, Eastern Ethiopia, from October 09–29, 2023. A self-administered questionnaire was utilized to collect the data. Epidata version 4.6 and SPSS Statistics version 27.0.1 were used for data entry and analysis, respectively. Both bivariable and multivariable logistic regression analyses were performed to identify the factors associated with breakfast consumption patterns. An adjusted odds ratio (AOR) with a 95% confidence interval (CI) was calculated to determine the strength of the association, and a p-value of 0.05 was used to determine statistical significance.

**Result:**

Nearly half, 46.2% (95% CI: 41.5, 51.4), of participants had irregular breakfast consumption (skipped). Being female (AOR = 5.28; 95% CI: 2.69, 10.36), family size of >5 (AOR = 4.76; 95% CI: 2.41, 9.36), being a rural resident (AOR = 3.34; 95% CI: 1.78, 6.25), no formal maternal education (AOR = 3.89; 95% CI: 2.09, 7.22), chewing khat (AOR = 3.13; 95% CI: 1.59, 6.16), cigarette smoking (AOR = 3.06; 95% CI: 1.02, 9.17), and eating disorders (AOR = 6.54; 95% CI: 2.19, 19.43) were significantly associated with irregular breakfast consumption patterns among adolescents.

**Conclusion:**

The findings of this study showed that the prevalence of irregular breakfast consumption (breakfast skipping) among adolescent high school students was high. Being female, rural residency, no formal maternal education, current smoking of cigarettes, current khat chewing, and eating disorders were identified as factors associated with breakfast consumption patterns. Given that almost half of adolescents in Tullo District skip breakfast, several modifiable factors associated with this practice, focused interventions are essential.

## Introduction

Breakfast is the primary and most important meal of the day which is considerably related to physiological, psychological, and social well-being. [1,2]. A healthy breakfast contains a balanced ratio of all the required nutrients for our body, and it should guarantee a median of 20–25% of the energy consumed throughout the day. [3]. Breakfast consumption improves attention, memory, and psychological performance. [4]. Eating breakfast is notably necessary during the adolescent period. Adolescents go through different biological, physical, and mental development processes. [5]. Moreover, during this period, adolescents have the greatest total energy requirement compared to any age group (~2,420 kcal/day) [6]. Therefore, breakfast consumption contributes approximately 24.3% to 27.8% of daily caloric intake, averaging around 680.7 kcal. [7].

Globally, the prevalence of breakfast skipping among adolescents ranged between 0.7% (in Japan) and 94% (in Portugal) [8–10]. In low- and middle-income countries (LMICs), the prevalence of breakfast skipping ranges between 23% and 38% [11,12]. Moreover, studies conducted in different parts of Ethiopia reported that breakfast skipping has ranged between 20% and 42% [4,13,14].

Missing breakfast is associated with risk factors for cardiac and metabolic health problems and is important in weight management. [15]. Similarly, several studies have shown that missing breakfast is related to worse lipid profiles, vital sign levels, endocrine resistance, and metabolic syndrome. [16]; increased risk of type 2 DM (diabetic mellitus) [17]; higher BMI (body mass index) [18]; and reduced performance in cognitive and psychosocial functions, as well as academic learning and achievement [13].

Breakfast consumption patterns are associated with sociodemographic, behavioral, and environmental factors. Sociodemographic factors include socioeconomic status, age, sex, and type of school. [19,20]. Environmental factors include eating or buying food prepared outside the home, maternal education and employment, and parental death. [11,19]. Breakfast skipping is significantly associated with an increased risk of type 2 DM. [17] poor academic performance, depression, lower happiness, posttraumatic stress disorder, loneliness, short and long sleep, sleep problems, restless sleep, and increasing levels of family income [21,22]. In Ethiopia, studies have shown that breakfast skipping among adolescents is associated with poorer academic performance. [4,13], and obesity [23]. Adolescents from low-income families and food-insecure households tend to skip their breakfast more than their counterparts; being female is also an important factor in skipping breakfast. [13]. This age group was chosen because, as they transition to adulthood, they are more likely to be left to set food plans, including breakfast consumption, than younger children [24].

Different scholars have intensively studied factors associated with breakfast consumption patterns, and the nutritional status of adolescents in Ethiopia [4,13,14]. However, there is a lack of clear data or insufficient studies on adolescent nutrition, in particular breakfast consumption patterns. Moreover, no study has been conducted particularly in this study area. Therefore, this study aimed to assess breakfast consumption patterns and associated factors among high school adolescent students in the Tullo district, Eastern Ethiopia.

## Methods and materials

### Study design and period

An institution-based cross-sectional study design was conducted. The study was conducted from October 09–29, 2023, in the Tullo district, Eastern Ethiopia. Tullo Woreda is one of the 17 Woredas of the West Hararghe Zone of the Oromia Region and is located 42 km from the zonal capital, Chiro, and 244 km from the capital of Addis Ababa. As per the 2007 population projection, the district has a total population of 147,384. The woreda covers 485.84 sq. km. The majority of the household’s income depends on small-scale cash cropping, mainly coffee (70.7%). According to the District Education office report, currently, there are four high schools with 3983 total high school students (Tullo woreda; Health office, Education office, and Agricultural office, 2021).

### Population and sampling

The source population for this study was all adolescents attending high schools in the Tullo District of Eastern Ethiopia. All randomly selected adolescents from the selected high schools during the study period in the Tullo District, Eastern Ethiopia, were the study population. All adolescents aged 14–19 years, registered during the academic year of 2023–2024 from selected high schools in the Tullo district, were included in the study, whereas students who had visual impairment and were critically ill during data collection were excluded from our study.

The required sample size was calculated by using a single population proportion formula. The proportion (p) of breakfast skipping is estimated to be 42.3% among adolescents from a previous study conducted on the assessment of breakfast eating habits and their association with cognitive performance of early adolescents [25]. By considering a 10% non-response rate, the sample size was estimated to be 414. We chose two out of the four public high schools in Tullo District using a simple random sampling method (lottery). A proportional stratified sampling technique was employed. The total sample size was allocated to the schools and sections proportional to the number of students in each selected school and section at the time of the study. The study participants were selected by a systematic sampling technique using the list of students enrolled in each school and section as a sampling frame. The sampling interval was determined by dividing the total number of students in the respective school grade level by the allocated sample size and was found to be five. The first participant was selected randomly by the lottery method, and then every fifth adolescent student was included in the study.

### Data collection and quality control

A pretested structured questionnaire was used to collect data in four sections: Section A on sociodemographic and socioeconomic status (SES), which were assessed using questions adapted from the Ethiopia Demographic and Health Survey 2016 report [26]; Section B on lifestyle factors like health status, smoking, alcohol use, and sleep. For assessment of eating disorders, the SCOFF(Sick, Control, One, Fat, Food) questionnaire validated among adolescents was used [27]. An adolescent having a score equal to or above 2 was considered to be at risk for eating disorders. Section C on breakfast patterns, including items consumed and reasons which was assessed using a standardized food frequency questionnaire [28]. There were a total of 16 food items or categories on the questionnaire; these items were then categorized into 9 main food groups by summing all the consumption frequencies of food items of the same group and recoding the value of each group above 7 as 7. Next, to construct new weighted food group scores, multiply the value acquired for each food group by its weight (Main Staples * 2, Pulses * 3, Vegetables * 1, Fruit * 1, Meat and Fish * 4, Milk * 4, Sugar * 0.5, Oil * 0.5, and Condiments * 0). The food consumption score was then calculated by adding the weighted food category scores and applying the appropriate thresholds (0–21 Poor, 21.5–35 Borderline, and > 35.5 Borderline) [29]. Section D on anthropometric measurements assessed participants’ nutritional status. The questionnaire was prepared in English and then translated into Afan Oromo and retranslated to English in order to check for consistency.

Data was collected by three BSC holder nurses fluent in the local language. The interview was conducted using a pretested questionnaire over 20 days. The process included self-reported responses and physical measurements. Weight and height scales were calibrated to ensure reliability. Students removed their shoes for weight, recorded to the nearest 0.1 kg, and stood barefoot for height, measured to the nearest 0.5 cm. Body mass index (BMI) was calculated (kg/m^2^), and WHO 2007 growth charts classified BMI-for-age-Z-score (BAZ) using z-scores for those aged 5–19 [30].

To maintain the quality and consistency of the data collection, two days of training were given to data collectors. This covered the objective of the study, procedures, and ethical issues. A pretest was conducted on 5% of the sample size outside the study area. Data collected were checked daily for completeness and consistency by the principal investigators. The scales were regularly checked and adjusted to zero after each measurement. To minimize measurement error, TEM (Technical Error of Measurement) was done before actual data collection with ten participants, and acceptable values for intra-evaluator and inter-evaluator were less than 1.5% and 2%, respectively.

### Operational definitions

#### Adolescents.

According to WHO definitions, adolescents are the age group between 10 and 19 years old [31].

#### Breakfast.

Defined as the first meal of the day, eaten before starting daily activities, before 10:00 am [13].

#### Breakfast consumption patterns.

Those who ate breakfast every day or six days in the 7 days preceding the survey were categorized as regular breakfast consumers, and those who skipped breakfast more than 2 days were categorized as breakfast skippers [4].

#### Food consumption score.

Used for household food security, calculated by examining the number of times certain foods, grouped into basic food groups, food consumed score was categorized as: (0–21) Poor; (21.5–35) Borderline; (>35) Acceptable. [29].

#### BMI.

The World Health Organization 2007 growth reference was used as a standard reference for classifying adolescents based on body mass index for age. BMI for age was classified as <−2 underweight, between −2 and 2 is normal, and >1 is overweight [30].

#### Family size.

Refers to the total number of people living in a house during the study period, and respondents are categorized as small family size (≤5) and large family size (>5) [13].

#### Eating disorder.

The questionnaire consists of five questions, and the response options (‘Yes’/‘No’) were scored by giving one point for a positive answer and zero points for a negative answer, and respondents who scored equal to or above 2 were considered to be at risk for eating disorders [27].

### Data analysis

After checking for completeness and consistency, the data were exported to SPSS Statistics version 27.0.1 for cleaning and analysis. Descriptive analysis was performed, and results were presented in tables, graphs, and charts. A bivariate logistic regression analysis was performed to assess associations between the dependent variable and the independent variables. The independent variables with p-values less than 0.25 in the bivariate analysis were considered for further analysis by multivariate logistic regression analysis to control for potential confounders and to detect the predictors of breakfast consumption patterns. Odds ratios, along with a 95% confidence interval, were estimated to measure the strength of the association. The level of statistical significance was declared at a P value less than 0.05. Multicollinearity was checked by calculating VIF, and model adequacy was checked by using the Hosmer and Lemeshow goodness of fit test.

### Ethics approval and informed consent

The study was conducted according to the Declaration of Helsinki. Ethical clearance was obtained from the Institutional Health Research Ethics Review Committee (IHRERC) of the College of Health and Medical Sciences, Haramaya University, with reference number IHRERC/181/2023. An official letter was written to the Tullo Woreda Education office for cooperation. A letter of permission was obtained from the Tullo Woreda Education office and selected high schools before the study was conducted. Informed, voluntary, written, and signed consent was obtained from the adolescent’s family for those less than 18 years of age and from students themselves if over 18 years old after informing them about the purpose, risks, and benefits of the study.

## Results

### Socio-demographic characteristics

From 414 eligible participants, 405 completed the study, yielding a response rate of 97.8%. More than half, 53.6%, of the participants were from grade 10, and 54.6% were female. The mean age of the study participants was 16.63, and the SD was 1.31. From all participants’ parents, 72.1% of the fathers and 42.2% of the mothers had attained some level of formal education (Table 1).

**Table 1:** Socio-demographic characteristics of adolescent high school students in the Tullo district, Oromia Region, Eastern Ethiopia, 2023.

Variables	Category	Frequency (405)	Percentage (%)
Age (in year)	Middle adolescent (14 –16 )	78	19.3
	Late adolescent (17 –19)	327	80.7
Sex	Female	221	54.6
	Male	184	45.4
Education level	Grade 9	188	46.4
	Grade 10	217	53.6
Religion	Orthodox	103	25.4
	Muslim	272	67.2
	Protestant	24	5.9
	Catholic	6	1.5
Residence			
	Urban	227	56.0
	Rural	178	44.0
Number of family members	≤5	150	37.0
	>5	255	63.0
With whom respondent live currently	Alone	39	9.6
	With friends	130	32.1
	With family	236	58.3
Mother’s marital status	Single	106	26.2
	Married	227	56.0
	Divorced	57	14.1
	Widowed	15	3.7
Mother’s educational status	No formal education	234	57.8
	Primary school	130	32.1
	Secondary school	18	4.4
	College and above	23	5.7
Father’s educational status	No formal education	113	27.9
	Primary school	120	29.6
	Secondary school	103	25.4
	College and above	69	17
Mother’s occupation	House wife	161	39.8
	Merchant	86	21.2
	Farmer	140	34.6
	Government employer	18	4.4
Father’s occupation	Government employer	53	13.1
	Merchant	86	21.2
	Farmer	262	64.7
	Others**	4	1.00
Average monthly income	>2000ETB	298	73.6
	< 2000ETB	107	26.4
Living arrangement	Alone	39	9.6
	With friends	130	32.1
	With family	236	58.3

Others**Private employee, NGO, pension

### Behavioral and medical characteristics

According to this study, 43.2% were currently khat chewers, 14.8% were cigarette smokers, and 5.9% consumed alcoholic drinks in the past 30 days. Regarding the time of wake-up, 37.0% of participants usually awoke before 6:00 am, and 2.7% of participants were awake after 8:00 a.m. Among the participants, 36.3% usually slept after 11:00 p.m., and 14.3% slept before 9:00 p.m. 12.1% of the total participants had an eating disorder or at least one symptom. Of those, 6.4% reported they make themselves sick because they feel uncomfortably full. The majority of respondents, 89.60%, walk to school, and only 10.4% use transport (Table 2).

**Table 2:** Behavioral and medical characteristics of adolescent high school students in Tullo district, Oromia Region, Eastern Ethiopia 2023.

Variables	Category	Frequency (405)	Percentage (%)
Current khat use	Yes	175	43.2
	No	230	56.8
Current cigarette smokers	Yes	60	14.8
	No	345	85.2
Current alcohol use	Yes	24	5.9
	No	381	94.1
Usual time of wake-up	Before 6:00 am	150	37.0
	6:00–7:00 am	103	25.4
	7:00–8:00 am	141	34.8
	After 8:00 am	11	2.7
Usual time of sleeping for the night	Before 9:00 pm	58	14.3
	9:00–10:00 pm	82	20.2
	10:00–11:00 pm	118	29.1
	After 11:00 pm	147	36.3
How do you come to the school	Walking	363	89.6
	Using transports	42	10.4
Having eating disorder	Yes	49	12.1
	No	356	87.9

### Nutritional characteristics

Based on nine food groups, a food consumption score was calculated, and 69.6% had food consumption that could be classified as acceptable. Based on the BMI for age, 16.8% were underweight and 80% were within the normal range. More than three-fourths, 77.5% of participants, never ate between meals during the day, and 66.40% of participants ate less than three meals a day (Table 3).

**Table 3:** Nutritional characteristics of adolescent high school students in Tullo district, Oromia Region, Eastern Ethiopia, 2023.

Variables	Category	Frequency (405)	Percentage (%)
Meal frequency	< 3times a day	269	66.4
	≥ 3times a day	136	33.6
Snack frequency	One times a day	63	15.6
	Two times a day	28	6.6
	Never	314	77.5
Food consumption score	Acceptable	282	69.6
	Borderline	86	21.2
	Poor	37	9.1
Nutritional status	Underweight	68	16.8
	Normal	324	80.0
	Overweight	13	3.2

### Breakfast consumption patterns

**Fig 1:**
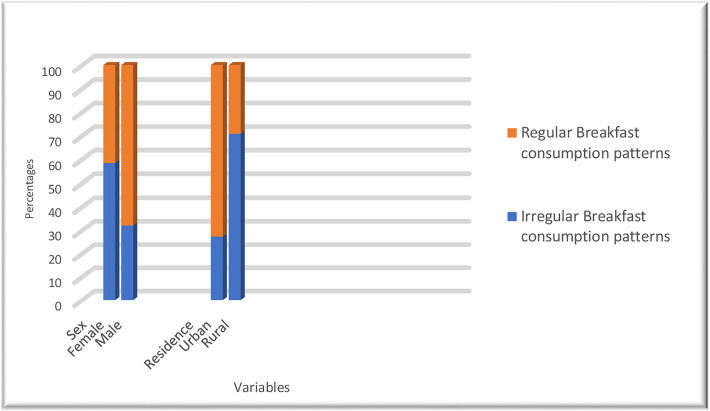
Breakfast consumption patterns with sex and residence variables among adolescent high school students in Tullo district, Oromia Region, Eastern Ethiopia, 2023.

With regard to breakfast consumption patterns, 46.2% skipped breakfast (95% CI: 41.5, 51.4), and 53.8% of adolescents regularly consumed breakfast. A chi-square test of independence to evaluate the relationship between sex and breakfast consumption pattern was significant, x^2^ (1, N = 405) = 27.00, p < 0.001. Females (57.9%) were more likely to skip breakfast than were males (32.1%). Also, the chi-square test of independence for residence and breakfast consumption pattern was significant, x^2^ (1, N = 405) = 73.92, p < 0.001. Being a rural resident (70.2%) was more likely to skip breakfast than were urban residents (27.3%) (Fig 1).

Among those who ate breakfast regularly, 67.4% ate it at home, with 55.1% of respondents’ mothers preparing their breakfast. According to the report by breakfast skippers (188), the most common reason for skipped breakfast was “they did not have enough time (29.7%)” followed by “poor appetite in the morning” (17.6%)”. The least common reason was (3.5%) “Don’t like to eat early” (Table 4).

**Table 4:** Breakfast characteristics of adolescent high school students in Tullo district, Oromia Region, Eastern Ethiopia, 2023.

Variables	Category	Frequency (n = 405)	Percentage (%)
Where do you usually eat your breakfast	From my home	273	67.4
	On the way to school	107	26.4
	Others*	25	6.2
Who prepares breakfast for you	Mother	223	55.1
	Myself	104	25.7
	Sister	51	12.6
	Others**	27	6.7
Families, teachers, or friends encourage you to have breakfast regularly.	Never	148	36.5
	Sometimes	84	20.7
	Often	45	11.1
	Always	128	31.6
Do you think it is important to eat breakfast?	Neutral	44	10.9
	Yes	283	69.9
	No	78	19.3
Reason for breakfast skipping	I don’t feel hungry	32	7.9
	No time to have breakfast	119	29.7
	I don’t find ready food to eat	34	8.4
	My family skip the breakfast and so I do	32	7.9
	Poor appetite in the morning	88	21.9
	I need to lose weight	26	6.5
	I don’t like to eat early	14	3.5
	Don’t have food to eat	56	14.0

*From school, From relative’s house, **Brother, Grandmother, Father, Aunts

### Factors associated with breakfast consumption patterns

In this study, variables such as sex, age, residence, family size, father’s educational status, mother’s educational status, father’s occupation, average monthly income of the family, BMI for age, food consumption score, current chew khat, current cigarette smoke, time of wake-up, and having an eating disorder were associated with breakfast consumption patterns at a p-value of 0.25 in bivariable analysis to identify candidate variables for multivariable analysis. In multivariate analysis, however, only being female, having a family size of >5, being a rural resident, having no formal education from the respondent’s mother, chewing khat, cigarette smoking, and eating disorders were significantly associated with breakfast consumption patterns among adolescents (Table 5).

**Table 5:** Factors associated with breakfast consumption patterns in adolescent high school students in Tullo district, Oromia region, Eastern Ethiopia 2023.

Variable		Breakfast consumption patterns		COR (95%CI)	AOR (95%CI)	P-value
		Irregular n (%)	Regular n (%)			
**Sex of student**	Female	128(58.4)	93(41.6)	2.92(1.94–4.39)	5.28(2.69–10.36)	<0.001**
	Male	59(32.1)	125(67.9)	1	1	
**Family size category**	>5 Persons	162(63.9)	93(36.1)	8.71(5.29–14.35)	4.76(2.41–9.36)	<0.001**
	≤5 Persons	25(16.7)	125(83.3)	1	1	
**Residence of student**	Rural	125(70.8)	53(29.2)	6.27(4.07–9.69)	3.34(1.78–6.25)	<0.001**
	Urban	62(27.3)	165(72.7)	1	1	
**Mother’s educational status**	No formal education	145(62.4)	89(37.6)	5.00(3.23–7.75)	3.89(2.09–7.22)	<0.001**
	Formal education	42(24.6)	129(75.4)	1	1	
**Current khat use**	Yes	115(66.3)	60(33.7)	4.21(2.76–6.39)	3.13(1.59–6.16)	<0.001**
	No	72(31.3)	158(68.7)	1	1	
**Current cigarette use**	Yes	53(88.3)	7(11.7)	11.92(5.26–26.99)	3.06(1.02–9.17)	0.046**
	No	134(38.8)	211(61.2)	1	1	
**Eating\disorder**	Yes	36(81.8)	13(22.2)	3.76(1.92–7.33)	6.54(2.19.–19.49)	<0.001**
	No	151(43.3)	205(56.7)	1	1	

**Significant at P-value < 0.05, COR = Crude Odd Ratio, AOR = Adjusted odd ratio, CI = Confidence interval.

Accordingly, female participants were 5.28 (AOR = 5.28; 95% CI: 2.69, 10.36) times more likely to skip breakfast than male participants. Participants who had > 5 family members were 4.76 (AOR = 4.76; 95% CI: 2.41–9.36) times more likely to skip breakfast than <5 family members. Rural residents were 3.34 (AOR = 3.34; 95% CI: 1.78, 6.25) times more likely to skip breakfast than urban residents. Participants’ mothers who never attained formal education were 3.89 (AOR = 3.89; 95% CI: 2.09, 7.22) times more likely to skip breakfast compared to those who attained primary and above school. There was a 3.13 (AOR = 3.13; 95% CI: 1.59, 6.16) times greater chance of skipping breakfast among those who chewed khat in the past 30 days than those who did not chew khat, and smoking cigarettes was associated with 4.12 times (AOR = 3.06; 95% CI: 1.02, 9.17) higher likelihood to skip breakfast than non-smokers. Of the participants who reported having an eating disorder or having one or more symptoms, they were 6.54 (AOR = 6.54; 95% CI: 2.19, 19.43) times more likely to skip breakfast than those who reported not having an eating disorder (Table 5).

## Discussion

According to this study, 46.2% (95% CI: 41.5, 51.4) of adolescent high school students skip breakfast. Our study found a consistent prevalence of breakfast skippers among study participants compared to other studies conducted in Ethiopia: in the North Shewa, Oromia Region, 41.3% [13], and in the Shebedino District, Southern Ethiopia, 42.3% [4]. This finding is also consistent with a comprehensive study conducted in more than 35 countries and a study conducted in India, which revealed that 45% [20] and 47.7% [32] of adolescents did not consume breakfast, respectively. Similarly, a study conducted in selected regions in Lebanon reported that the prevalence of breakfast skipping was 42.8% [33]. Whereas this study found a relatively higher prevalence of breakfast skippers compared to studies conducted in Mekelle City, Ethiopia (2019) revealed that among public and private secondary schools, from 853 total participants, 244 (28.6%) did not eat breakfast daily; among this group, 36.6% and 20% reported that breakfast intake was not eaten daily, respectively [14] and in Nigeria, 23% [12] of them had not eaten breakfast daily; in Palestine, 38% [11] and, in Jordan, 18.5% [34].

Regarding reasons for skipping breakfast meals, the study revealed that more than one fourth (29.7%) of the students skipped breakfast because they “don’t have time to eat breakfast, followed by (21.9%) “poor appetite in the morning. These common reasons for skipping breakfast have also been reported in previous studies conducted in Ethiopia and other countries [11,13,34]. The reason for this finding may be many students chew khat to study at night and are often rushing to class since they have little time for breakfast in the morning. It has been reported that skipping breakfast has been a means of saving time by most adolescents in the morning to get to school on time [35].

In this study, female participants were 5.28 times more likely to skip breakfast than male participants. This finding is in line with a study conducted in North Shewa, Ethiopia [13], and similarly other studies also revealed that female participants were more likely to skip breakfast than males [19,36]. This could be because of the females are probably more self-conscious about their weight and/or appearance, which makes them more prone to engage in weight-controlling activities like skipping breakfast [37]. Similarly, in some cultures, women are more likely to prioritize family needs over their own, leading to less attention to personal health and nutrition, including skipping breakfast and women are more likely to eat in response to emotional states and social contexts, which can lead to irregular meal patterns, including skipping breakfast [38,39].

Additionally, rural residents were 3.34 times more likely to skip breakfast as compared to those who lived in urban areas. This result is in line with a study conducted in Jenin governance, West Bank, where the participants who lived in rural areas had 1.26 times the risk of always skipping breakfast compared to those living in major cities [11]., This could be due to the reason that rural environments often have different cultural norms and attitudes towards food compared to urban areas, and in some urban settings, there may be a greater emphasis on regular mealtimes and the importance of nutrition, which could influence individuals’ patterns and reduce the likelihood of skipping breakfast [37]. Additionally, rural students often face food insecurity and limited access to nutritious meals [40].

The current study also revealed that higher maternal education would improve regular breakfast consumption patterns. Participants whose mothers had no formal education were 3.89 times more likely to skip breakfast than those whose mothers had primary and above education. This result is in line with the study conducted in the Shebedino district, southern Ethiopia, and in Jidda, Saudi Arabia [19]. This is explained by mothers with no formal education, who may have limited knowledge about nutrition and healthy eating habits, and limited financial resources may result in food insecurity within the household, leading to breakfast skipping [41]. Additionally, higher maternal education levels lead to increased nutritional knowledge, enabling mothers to make informed dietary choices for their children and help them to understand the importance of regular breakfast consumption, leading to healthier eating habits in their children [42,43].

In this study, current smokers were 3.06 times more likely to skip breakfast compared to nonsmokers. This is consistent with the study conducted in Bangladesh [44]. A possible explanation for the correlation between smoking and skipping breakfast is that smoking can decrease appetite, leading to lower breakfast consumption [45]. However, our finding is inconsistent with a study conducted to assess breakfast consumption and its socio-demographic and lifestyle correlates in school children in 41 countries participating in the HBSC study [37].

Moreover, in this study, current khat chewers are 3.13 times more likely to skip breakfast than those who do not chew khat currently. This result is supported by research done on factor analysis–eating patterns among khat chewers in Saudi Arabia [24]. The possible explanation for this could be khat is often consumed in social settings as a communal activity that can alter eating patterns, such as skipping meals like breakfast, and its normalization among peers, particularly in educational institutions, reinforces its consumption while overshadowing traditional meal times [46,47]. In addition to this, individuals who chew khat may be less likely to make healthy choices regarding their diet, including skipping breakfast, and chewing khat may disrupt daily routines, including regular meal times [24].

The other variable that had an association with breakfast consumption pattern was eating disorder. Furthermore, this study revealed that breakfast skipping was 6.54 times more common among those who reported having the risk of an eating disorder than those who did not have. This study was supported by research conducted in the USA and high-income countries, which describes that during adolescence, regular breakfast consumption was negatively associated with an eating disorder [41,48,49]. Which can be described as the likelihood of developing any form of eating disorder was diminished by eating breakfast [50]. The possible explanation for this is that adolescents at risk for eating disorders may skip breakfast as a means of calorie restriction or due to body dissatisfaction, and the disruption in family meal practices can also lead to increased breakfast skipping, particularly among adolescents who prioritize autonomy in food choices [51]

This study is not without limitations. Since some questions asked about events that occurred four weeks before the study period, recall bias might be introduced into study; for this, sufficient time to think and answer questions and used neutral interviewers. Social desirability bias was also another limitation, since some students might report what they think is socially acceptable or favorable rather than their true experiences. This may lead to overestimation or understatement of certain behaviors. Self-administered questionnaires were used and confidentiality was ensured to minimize this bias.

## Conclusion

The finding of this study showed that the prevalence of irregular breakfast consumption (breakfast skipping) among adolescent high school students was high. Being female, rural residency, no formal maternal education, smoking of cigarettes, khat chewing, and eating disorders were identified as factors associated with breakfast consumption patterns. Based on this findings, school-based health education initiatives highlighting the importance of regular breakfast intake, especially among girls and urban people is recommended. Interventions must focus on educating mothers and discouraging unhealthy behaviors like smoking and chewing khat. Additionally, schools should promote eating problem screening and support and coordinated implementation of these strategies can increase breakfast intake among adolescents, which may benefit their academic and health outcomes. Further research that uses mixed study designs is recommended to understand in depth the breakfast consumption patterns and the culture surrounding it, including perceptions towards breakfast consumption patterns among adolescents.

## Supporting information

S1 File (XLS)

## Abbreviations

AOR: Adjusted Odds Ratio
BAZ: BMI-for-age-Z-score
BMI: Body Mass Index
CDC: Center for Disease Control
CED: Chronic Energy Deficiency
CSA: Central Statistical Agency
EDHS: Ethiopian Demographic Health Survey
FANTA: Food and Nutrition Technical Assistance
GBD: Global Disease Burden
HBSC: Health Behavior in School-aged Children
IHRERC: Institutional Health Research Ethics Review Committee
MUAC: Mid Upper Arm Circumference
NGO: Non-Governmental Organization
SCOFF: Sick, Control, One, Fat, Food
SS: Systematic sampling
T2DM: Type 2 diabetes mellitus
TEM: Technical Error of Measurement
UNICEF: United Nations International Children’s Emergency Fund
WFP: World Food Program
WHO: World Health Organization

## References

[1] Chen J, Cheng J, Liu Y, Tang Y, Sun X, Wang T, et al. Associations between breakfast eating habits and health-promoting lifestyle, suboptimal health status in Southern China: a population based, cross sectional study. J Transl Med. 2014;12:348. doi: 10.1186/s12967-014-0348-1 25496597 PMC4269950

[2] Garcia-Serrano AM, Duarte JMJ. Brain metabolism alterations in type 2 diabetes: what did we learn from diet-induced diabetes models?. Fin. 2020;14:229. 10.3389/fnins.2020.00229 PMC7101159 32265637

[3] Ortega R, Requejo AM, Redondo R, López‐Sobaler A, Andrés P, Ortega A. Breakfast habits of different groups of Spanish schoolchildren. Journal of Human Nutrition and Dietetics. 1996;9(1):33–41.

[4] Adole AA, Ware MB. Assessment of breakfast eating habits and its association with cognitive performance of early adolescents (11-13 years) in Shebedino District, Sidama Zone, Southern Ethiopia. J Food Nutr Sci. 2014;2(4):130–7.

[5] Rani R, Dharaiya CN, Singh B. Importance of not skipping breakfast: A review. International Journal of Food Science & Technology. 2021;56(1):28–38.

[6] Ferrari GLdM, Kovalskys I, Fisberg M, Gomez G, Rigotti A, Sanabria LYC, et al. Anthropometry, dietary intake, physical activity and sitting time patterns in adolescents aged 15–17 years: An international comparison in eight Latin American countries. BMC pediatrics. 2020;20:1–16. 31964386 10.1186/s12887-020-1920-x PMC6971876

[7] de Rufino Rivas P, Redondo Figuero C, Amigo Lanza T, González-Lamuño D, García Fuentes M, Grupo AVENA. Breakfast and snack of schooled adolescents in Santander. Nutr Hosp. 2005;20(3):217–22. 15989069

[8] Sun Y, Sekine M, Kagamimori S. Lifestyle and overweight among Japanese adolescents: the Toyama Birth Cohort Study. J Epidemiol. 2009;19(6):303–10. doi: 10.2188/jea.je20080095 19776497 PMC3924099

[9] Mota J, Fidalgo F, Silva R, Ribeiro JC, Santos R, Carvalho J, et al. Relationships between physical activity, obesity and meal frequency in adolescents. Ann Hum Biol. 2008;35(1):1–10. doi: 10.1080/03014460701779617 18274921

[10] Souza MR, Neves MEA, Gorgulho BM, Souza AM, Nogueira PS, Ferreira MG, et al. Breakfast skipping and cardiometabolic risk factors in adolescents: Systematic review. Rev Saude Publica. 2021;55:107. doi: 10.11606/s1518-8787.2021055003077 34932697 PMC8664063

[11] Badrasawi M, Anabtawi O, Al-Zain Y. Breakfast characteristics, perception, and reasons of skipping among 8th and 9th-grade students at governmental schools, Jenin governance, West Bank. BMC Nutr. 2021;7(1):42. doi: 10.1186/s40795-021-00451-1 34353371 PMC8342035

[12] Lateef OJ, Njogu E, Kiplamai F, Haruna US, Lawal RA. Breakfast, Food Consumption Pattern and Nutritional Status of Students in Public Secondary Schools in Kwara State, Nigeria. Pakistan J of Nutrition. 2016;15(2):140–7. doi: 10.3923/pjn.2016.140.147

[13] Feye D, Gobena T, Brewis A, Roba KT. Adolescent breakfast skipping is associated with poorer academic performance: a school-based study from Hidhabu Abote District, Ethiopia. J Health Popul Nutr. 2023;42(1):79. doi: 10.1186/s41043-023-00424-z 37568241 PMC10422701

[14] Andargie M, Gebremariam K, Hailu T, Addisu A, Zereabruk K. Magnitude of Overweight and Obesity and Associated Factors Among Public and Private Secondary School Adolescent Students in Mekelle City, Tigray Region, Ethiopia, 2019: Comparative Cross-Sectional Study. Diabetes Metab Syndr Obes. 2021;14:901–15. doi: 10.2147/DMSO.S262480 33688225 PMC7936680

[15] Mohiuddin A. Skipping breakfast everyday keeps well-being away. Acta Medica. 2019;50(1):26–33.

[16] Monzani A, Ricotti R, Caputo M, Solito A, Archero F, Bellone S, et al. A Systematic Review of the Association of Skipping Breakfast with Weight and Cardiometabolic Risk Factors in Children and Adolescents. What Should We Better Investigate in the Future?. Nutrients. 2019;11(2):387. doi: 10.3390/nu11020387 30781797 PMC6412508

[17] Bi H, Gan Y, Yang C, Chen Y, Tong X, Lu Z. Breakfast skipping and the risk of type 2 diabetes: a meta-analysis of observational studies. Public Health Nutr. 2015;18(16):3013–9. doi: 10.1017/S1368980015000257 25686619 PMC10271832

[18] Ma X, Chen Q, Pu Y, Guo M, Jiang Z, Huang W, et al. Skipping breakfast is associated with overweight and obesity: A systematic review and meta-analysis. Obes Res Clin Pract. 2020;14(1):1–8. doi: 10.1016/j.orcp.2019.12.002 31918985

[19] Al-Hazzaa HM, Alhowikan AM, Alhussain MH, Obeid OA. Breakfast consumption among Saudi primary-school children relative to sex and socio-demographic factors. BMC Public Health. 2020;20(1):448. doi: 10.1186/s12889-020-8418-1 32252722 PMC7132954

[20] Currie C, Roberts C, Settertobulte W, Morgan A, Smith R, Samdal O. Young people’s health in context: Health behaviour in school-aged children (HBSC) study: international report from the 2001/2002 survey. Regional Office for Europe: World Health Organization. 2004.

[21] Huang C-J, Hu H-T, Fan Y-C, Liao Y-M, Tsai P-S. Associations of breakfast skipping with obesity and health-related quality of life: evidence from a national survey in Taiwan. Int J Obes (Lond). 2010;34(4):720–5. doi: 10.1038/ijo.2009.285 20065977

[22] Terry AL, Wambogo E, Ansai N, Ahluwalia N. Breakfast intake among children and adolescents: United States, 2015–2018. 2020. 33054919

[23] Kidus B. The effect of change management on employee performance: In case of Ethio Telecom North region at Debre Birhan town. 2021.

[24] Alsayegh AA, Chandika RM, Tubaigi AA, Majrashi AM, Meree WA, Asiri AA. Factor analysis - Eating patterns among khat chewers. J Family Med Prim Care. 2022;11(6):2774–9. doi: 10.4103/jfmpc.jfmpc_1924_21 36119204 PMC9480645

[25] Feye D, Gobena T, Brewis A, Roba KT. Adolescent breakfast skipping is associated with poorer academic performance: a school-based study from Hidhabu Abote District, Ethiopia. J Health Popul Nutr. 2023;42(1):79. doi: 10.1186/s41043-023-00424-z 37568241 PMC10422701

[26] CSA a I. Ethiopia demographic and health survey 2016. Addis Ababa, Ethiopia: CSA and ICF. 2017.

[27] Hautala L, Junnila J, Alin J, Grönroos M, Maunula A-M, Karukivi M, et al. Uncovering hidden eating disorders using the SCOFF questionnaire: cross-sectional survey of adolescents and comparison with nurse assessments. Int J Nurs Stud. 2009;46(11):1439–47. doi: 10.1016/j.ijnurstu.2009.04.007 19446810

[28] Brotherton AM. Principles of nutritional assessment. Wiley Online Library. 2006.

[29] Wiesmann D, Bassett L, Benson T, Hoddinott J. Validation of the world food programme s food consumption score and alternative indicators of household food security. Intl Food Policy Res Inst. 2009.

[30] WHO. Growth reference 5-19 years - BMI-for-age (5-19 years). World Health Organization. 2007.

[31] WHO. Technical consultation on indicators of adolescent health: global reference list of health indicators for adolescents (aged 10-19 years). Geneva, Switzerland: World Health Organization. 2016.

[32] Aarthi M, Priya VV, Gayathri RGR. Awareness on the effects of skipping breakfast among adolescents. Drug Invention Today. 2018;10:998–1001.

[33] Chami ME, Sacre Y, Matta J. The prevalence of breakfast skipping and its association with lifestyle factors and weight in 11-15 years adolescents from selected Lebanese regions. Occupational Medicine and Health Affairs. 2017;5:1–10.

[34] Bashtawy A. Breakfast eating habits among schoolchildren. J Pediatr Nurs. 2017;36:118–23. 28888491 10.1016/j.pedn.2017.05.013

[35] Buxton CNA. Ghanaian junior high school adolescents dietary practices and food preferences: implications for public health concern. J Nutrition & Food Sciences. 2014;4(5):1.

[36] Fiúza RF d P, Muraro AP, Rodrigues PRM, Sena E d MS, Ferreira MG. Omissão do desjejum e fatores associados entre adolescentes brasileiros. Revista de Nutrição. 2017;30(5).

[37] Vereecken C, Dupuy M, Rasmussen M, Kelly C, Nansel TR, Al Sabbah H, et al. Breakfast consumption and its socio-demographic and lifestyle correlates in schoolchildren in 41 countries participating in the HBSC study. Int J Public Health. 2009;54 Suppl 2(Suppl 2):180–90. doi: 10.1007/s00038-009-5409-5 19639257 PMC3408388

[38] Smith KJ, McNaughton SA, Cleland VJ, Crawford D, Ball K. Health, behavioral, cognitive, and social correlates of breakfast skipping among women living in socioeconomically disadvantaged neighborhoods. J Nutr. 2013;143(11):1774–84. doi: 10.3945/jn.113.181396 23986365

[39] Grzymisławska M, Puch EA, Zawada A, Grzymisławski MJA, Medicine E. Do nutritional behaviors depend on biological sex and cultural gender?. 2020;29(1). 10.17219/acem/111817 32017478

[40] Nanney MS, Shanafelt A, Wang Q, Leduc R, Dodds E, Hearst M, et al. Project BreakFAST: Rationale, design, and recruitment and enrollment methods of a randomized controlled trial to evaluate an intervention to improve School Breakfast Program participation in rural high schools. Contemp Clin Trials Commun. 2016;3:12–22. doi: 10.1016/j.conctc.2015.12.009 27141531 PMC4850496

[41] Fadare O, Amare M, Mavrotas G, Akerele D, Ogunniyi A. Mother’s nutrition-related knowledge and child nutrition outcomes: Empirical evidence from Nigeria. PLoS One. 2019;14(2):e0212775. doi: 10.1371/journal.pone.0212775 30817794 PMC6394922

[42] van Ansem WJ, Schrijvers CT, Rodenburg G, van de Mheen D. Maternal educational level and children’s healthy eating behaviour: role of the home food environment (cross-sectional results from the INPACT study). Int J Behav Nutr Phys Act. 2014;11:113. doi: 10.1186/s12966-014-0113-0 25212228 PMC4177694

[43] Gebremariam MK, Henjum S, Hurum E, Utne J, Terragni L, Torheim LE. Mediators of the association between parental education and breakfast consumption among adolescents : the ESSENS study. BMC Pediatr. 2017;17(1):61. doi: 10.1186/s12887-017-0811-2 28228124 PMC5322630

[44] Biswas S, Alam SS, Sayem AH, Hossain M, Mithu SH, Akther S. Breakfast skipping and associated factors: experience from students at public university in Noakhali district, Bangladesh. Hindu. 2020;40:9.

[45] Gregersen NT, Møller BK, Raben A, Kristensen ST, Holm L, Flint A, et al. Determinants of appetite ratings: the role of age, gender, BMI, physical activity, smoking habits, and diet/weight concern. Food Nutr Res. 2011;55:10.3402/fnr.v55i0.7028. doi: 10.3402/fnr.v55i0.7028 21866221 PMC3160809

[46] Birhan TA, Worku W, Azanaw J, Yohannes L. Khat chewing practice and associated factors among medical students in university of Gondar, Ethiopia, 2019: a cross-sectional study. 2020. 10.1177/1178221821999079 PMC7930654 33716504

[47] Wood EA, Stark H, Case SJ, Sousa B, Moreno M, Motuma A, et al. Khat use and related determinants among pregnant women within Haramaya, Ethiopia: a mixed methods study. Front Glob Womens Health. 2024;5:1359689. doi: 10.3389/fgwh.2024.1359689 38784944 PMC11112567

[48] Sliwa SAJ. Skipping breakfast and academic grades, persistent feelings of sadness or hopelessness, and school connectedness among high school students—Youth Risk Behavior Survey, United States, 2023. Morb Mortal Wkly Rep. 2024;73. 10.15585/mmwr.su7304a10 PMC11559677 39378262

[49] Daneshvar MJ. Breakfast skipping as an indicator of unhealthy lifestyle among adolescents: A short letter. 2023;2023:2023.04. https://doi.org/10.23288359

[50] Agüera Z, Sánchez I, Granero R, Riesco N, Steward T, Martín-Romera V, et al. Short-Term Treatment Outcomes and Dropout Risk in Men and Women with Eating Disorders. Eur Eat Disord Rev. 2017;25(4):293–301. doi: 10.1002/erv.2519 28474473

[51] Holtzman NS. To skip or not to skip? Varying definitions of breakfast skipping and associations with disordered eating, obesity, and depression. 2010.

