# Supplementary material for: Breakfast consumption patterns and associated factors among adolescent high-school students in Tullo District, Eastern Ethiopia
Source: PLoS One. 2025 Aug 5;20(8):e0329608. doi: 10.1371/journal.pone.0329608 (PMC12324110; doi:10.1371/journal.pone.0329608)
Supplement: Attachment — Submitted filename: Response to Reviewers.pdf [file pone.0329608.s003.pdf]

Date: June, 2025

Dear editorial board members (s) of the PLOS ONE journal

The authors have been recalled to revise the manuscript entitled **"Breakfast Consumption Patterns and Associated Factors among Adolescent High-School Students in Tullo District, Eastern Ethiopia" under** the **PLOS ONE** journal, which was submitted for publication. So, we received the editor's and reviewer's revision comments to improve the manuscript before its publication.

Thank you for receiving comments, suggestions, directions, and questions from the editor and reviewer. As we said, we are very happy to receive constructive and valuable comments that will improve the manuscript. Accordingly, we have considered all the comments, questions, directions, and suggestions and provided a point-by-point response letter.

Finally, we have submitted all the required documents in their revised form. We hope that we have addressed all the suggestions, directions, and raised questions, and if you believe that the point(s) are not addressed, please let us know.

Thank you very much to all editors (s), and reviewers

On behalf of the authors

Yours sincerely,

Correspondence author

Point-by-point response letter

Reviewer 1 comments

The study aimed to assess breakfast consumption patterns and their associated factors among adolescent high school students in the Tullo district, Eastern Ethiopia.
Overall, the study achieved its objectives, the methods followed are sound and the results interpretation is good. There is few points to address in order to achieve perfection.

1.In the discussion, part it would be interesting to compare findings of this study with different populations of different age groups. with population of different socio-economic background and discuss the difference or similarities.

Authors reply: Thank you very much for your important comment and suggestions on improving the manuscript. Since skipping breakfast consumption is not significantly associated with age variable we couldn’t discuss it with population age group.

2. It would be also interesting to highlight in the discussion the implications of these findings. in other terms knowing these % and associations what do we conclude or how this will help us, policy makers and other stakeholders. Overall, what is the advantage of knowing these findings.

Authors reply: Thank you very much for your important comment and suggestions on improving the manuscript. Based on your comment, we made a correction. Look at revised manuscript.

2.It would be great if you can associate these data with the school grades to see if there is any impact of skipping breakfast on school performance as mentioned in the abstract. check the impact on health status, IQ. Schools should have records for the assessed population.

Authors reply: Thank you very much for your important comment and suggestions on improving the manuscript. Since skipping breakfast consumption is not significantly associated with school grades, and school performance (IQ)we couldn’t discuss it.

3.In the discussion and conclusion, the authors used "we" rephrase in passive tense.

Authors reply: Thank you very much for your important comment and suggestions on improving the manuscript. Based on your comment, we made a correction. Look at revised manuscript.

4.Table 1 Education level % there is a"the" to be deleted. in the tables add? when you are quantifying a question

<u>Authors reply</u>: Thank you very much for your important comment and suggestions on improving the manuscript. Based on your comment, we made a correction. Look at revised manuscript.

5.Table 4 "myself" one word. add ?

<u>Authors reply</u>: Thank you very much for your important comment and suggestions on improving the manuscript. Based on your comment, we made a correction. Look at revised manuscript.

6.Figure to change the format, letter font and size. they are very blurr

<u>Authors reply</u>: Thank you very much for your important comment and suggestions on improving the manuscript. Based on your comment, we made a correction. Look at revised manuscript.

Reviewer 2 comments

Dear Authors,

The manuscript entitled "Breakfast Consumption Patterns and Associated Factors among Adolescent High School Students in Tullo District, Eastern Ethiopia, addresses an important public health issue in a relatively under-researched context—adolescent nutrition in rural Ethiopia. The focus on breakfast consumption among high school students provides valuable insights into adolescent dietary behavior, which has strong links to educational performance and health outcomes. The research objective is well-articulated and grounded in existing literature gaps. The authors made a strong case for the need to assess breakfast habits in the Tullo District due to a lack of local data.

<u>Authors reply</u>: Thank you very much for your POSITIVE and Energetic feedback the manuscript.

1. There are numerous grammatical errors and awkward phrasings throughout the manuscript. For instance, phrases like "There is growing proof to recommend..." should be revised to "There is growing evidence suggesting...".

<u>Author's reply</u>: Thank you very much for your important comment and suggestions on improving the manuscript. Based on your comment, we corrected. Look at the revised manuscript.

2. A professional language edit is highly recommended to enhance readability and clarity.

<u>Author's reply</u>: Thank you very much for your important comment and suggestions on improving the manuscript. Based on your comment, we corrected. Look at the revised manuscript.

3. There is an inconsistency in stating "being female, urban residency…" as risk factors in the conclusion when rural residency was actually associated with higher breakfast skipping. This needs correction to avoid misinterpretation.

<u>Author's reply</u>: Thank you very much for your important comment and suggestions on improving the manuscript. Based on your comment, we corrected. Look at the revised manuscript.

4. While the study finds associations, the cross-sectional design precludes any causal inference. The authors should be more cautious in statements like "causes breakfast skipping" and instead say "is associated with."

<u>Author's reply</u>: Thank you very much for your important comment and suggestions on improving the manuscript. Based on your comment, we corrected. Look at the revised manuscript.

5. The discussion, though comparative, would benefit from a deeper theoretical exploration of why some factors (e.g., maternal education, khat use) are linked to breakfast skipping. Including more culturally grounded explanations could enrich the discussion.

<u>Author's reply</u>: Thank you very much for your important comment and suggestions on improving the manuscript. Based on your comment, we corrected. Look at the revised manuscript.

6. The classification for BMI is inconsistent; underweight is described as <-2, and overweight as >1. This appears incorrect based on the WHO BAZ classification. Please clarify or correct this to avoid misclassification bias.

<u>Author's reply</u>: Thank you very much for your important comment and suggestions on improving the manuscript. Based on the WHO BAZ classification, it is a correct classification using Z-score.

7. Tables are overly long and could benefit from simplification and clearer formatting. Consider merging or streamlining some tables for better presentation.

<u>Author's reply</u>: Thank you very much for your important comment and suggestions on improving the manuscript.

8. Figures were not included in the review copy. Please ensure they are properly embedded and described within the manuscript.

<u>Author's reply</u>: Thank you very much for your important comment and suggestions on improving the manuscript. Based on your comment, we corrected. Look at the revised manuscript.

9. Some of the citations are outdated or not peer-reviewed. The study would benefit from including more recent, high-impact studies, especially from Sub-Saharan Africa or other LMIC contexts.

<u>Author's reply</u>: Thank you very much for your important comment and suggestions on improving the manuscript. Based on your comment, we corrected. Look at the revised manuscript.

10. The manuscript lacks consideration for other potential confounders such as dietary diversity, access to food, school schedule, or mental health beyond eating disorders.

<u>Author's reply</u>: Thank you very much for your important comment and suggestions on improving the manuscript.

11. Define all abbreviations at first use in the abstract and main text (e.g., AOR, FFQ, BAZ).

<u>Author's reply</u>: Thank you very much for your important comment and suggestions on improving the manuscript. Based on your comment, we corrected. Look at the revised manuscript.

12. Clarify why certain cut-offs (like ≥6 times per week for regular breakfast consumption) were chosen.

Author's reply: Thank you very much for your important comment and suggestions on improving the manuscript. Cut-offs like "≥6 times per week for regular breakfast consumption" were used since, as a rule of thumb, at least one person should consume breakfast six times per week to say no skipping.

13. The conclusion should be more concise and avoid repetition from earlier sections.

Author's reply: Thank you very much for your important comment and suggestions on improving the manuscript. Based on your comment, we corrected. Look at the revised manuscript.

Reviewer 3 comments

Reviewer #3: This is a cross-sectional study, conducted in Eastern Ethiopia with a sample of 405 adolescents aged 14-19 years, regarding breakfast eating patterns and its associated factors. Of participants, 46% skipped breakfast, called as "irregular breakfast consumption", which was significantly associated with being female, having family size >5 members, living in the rural area, no formal maternal education, chewing khat, smoking and presenting eating disorders. The study is well described, and detailed, the analysis seems adequate and well conducted. However, major revision is recommended to improve consistency.

Tables should be revised to provide clearer information for the reader.

Authors reply: Thank you very much for your important comment and suggestions on improving the manuscript. Based on your comment, we made a correction. Look at revised manuscript.

Recommendations:

English review is recommended.

Authors reply: Thank you very much for your important comment and suggestions on improving the manuscript. Based on your comment, we made a correction. Look at revised manuscript.

- Abstract:

o Page 2, lines 22-24: "there is a dearth of evidence on the comprehensive understanding of the

breakfast consumption patterns. This study aimed to assess breakfast consumption patterns”. Is this the main aim of the study? Throughout the manuscript there is much more emphasis on the associated factors analysis, besides, there seems to be a good body of evidence on the literature regarding breakfast consumption, how does this study advances in relation to the already existing evidence?

Authors reply: Thank you very much for your important comment and suggestions on improving the manuscript. Based on your comment, we made a correction. Look at revised manuscript. This study is focused on breakfast consumption patterns and factors associated with it.

o Page 2, lines 28-29: the sentence “The data were entered into Epidata version 4.6 and exported to SPSS Statistics version 27.0.1 for analysis” is not essential for the abstract section and can be subtracted.

Authors reply: Thank you very much for your important comment and suggestions on improving the manuscript. Based on your comment, we made a correction. Look at revised manuscript.

o Page 3, line 43: it is written “urban residency”, however in line 36 it was mentioned as rural residency. Furthermore, in the conclusion section of the abstract there is no need to cite all associated factors one by one, the authors can summarize findings and find the most important message of the study, highlighting why these results matter for the literature.

Authors reply: Thank you very much for your important comment and suggestions on improving the manuscript. Based on your comment, we made a correction. Look at revised manuscript.

- Introduction:

o When citing existing evidence of other studies regarding associated factors to breakfast eating patterns, bring more context about the studies, where was it conducted, what is the sample size, the age of the individuals, the year the data were collected, and the magnitude of association being compared?

Authors reply: Thank you very much for your important comment and suggestions on improving the manuscript.

o Page 3, lines 52-53: “and it should guarantee a median of 20-25% of the energy consumed throughout the day” is this information relevant for the study aims and findings?

<u>Authors reply</u>: Thank you very much for your important comment and suggestions on improving the manuscript. We hope this study is relevant for knowing how much the breakfast consumption affects our performance if we miss it.

o Page 3, lines 56-57: “Moreover, during this period adolescents have the greatest total energy requirement compared to any age group (~2,420 kcal/day)” this information seemed a bit floaty from the rest of the paragraph, it would benefit from further exploring how this information is related to the importance of eating breakfast during this period of life.

<u>Authors reply</u>: Thank you very much for your important comment and suggestions on improving the manuscript. Based on your comment, we made a correction. Look at revised manuscript.

o Page 3, line 58: in which countries were observed the lowest and highest breakfast skipping prevalence cited?

<u>Authors reply</u>: Thank you very much for your important comment and suggestions on improving the manuscript. Based on your comment, we made a correction. Look at revised manuscript.

o Page 3, line 59: usually it is written as “low- and middle-income countries”.

<u>Authors reply</u>: Thank you very much for your important comment and suggestions on improving the manuscript. Based on your comment, we made a correction. Look at revised manuscript.

o Page 4, lines 79-81: “Different scholars have intensively studied factors associated with breakfast eating habits and the nutritional status of adolescents in Ethiopia (4, 10, 11). However, there is a lack of clear data or insufficient studies on adolescent nutrition in particular breakfast consumption patterns” what is being considered as the difference between “breakfast eating habits” and “breakfast consumption patterns”? The phrasing is confusing and do not depict well enough how this study advances in regards of the existing literature on the topic.

<u>Authors reply</u>: Thank you very much for your important comment and suggestions on improving the manuscript. Based on your comment, we made a correction. Look at revised

manuscript.

o Page 4, lines 82-83: “Moreover, breakfast consumption patterns data was not included in the Ethiopia Demographic and Health Survey (21)” why is this information important?

Authors reply: Thank you very much for your important comment and suggestions on improving the manuscript. Based on your comment, we made a correction. Look at revised manuscript.

- Methods

o Congratulate to the authors for the well described methods.

o Page 7, line 144: describe what the acronym “TEM” means.

Authors reply: Thank you very much for your important comment on improving the manuscript. Based on your comment, we made a correction. Look at revised manuscript.

o Page 9, line 176: describe what the acronym “VIF” means.

Authors reply: Thank you very much for your important comment on improving the manuscript. Based on your comment, we made a correction. Look at revised manuscript.

- Results

o Page 9, lines 185-186: “According to this study, 43.2% were currently khat chewers, 14.8% were cigarette smokers, and 5.9% consumed alcoholic drinks in the past 30 days” are these data from adolescents or the parents?

Authors reply: Thank you very much for your important comment on improving the manuscript. This data is from the adolescents.

o Table 3: improvement in description of each variable and category is recommended. “Food frequency” of some specific food group, or meal frequency, for the category: 3 times a day, a week?

Authors reply: Thank you very much for your important comment on improving the manuscript. Based on your comment, we made a correction. Look at revised manuscript.

o Table 5: the table would benefit from providing an overall row about frequency and percentage of occurrence for each breakfast consumption pattern category.

Authors reply: Thank you very much for your important comment on improving the manuscript. Based on your comment, we made a correction. Look at revised manuscript.

- Discussion

o When citing existing evidence of other studies, bring more context about them, what is the study design, where was it conducted, what is the sample size, the age of the individuals, the year the data were collected, etc., so the reader can compare this study and the cited study?

Authors reply: Thank you very much for your important comment on improving the manuscript. Based on your comment, we made a correction. Look at revised manuscript.

o Page 13, line 276: there seems to be a typo.

Authors reply: Thank you very much for your important comment on improving the manuscript. Based on your comment, we made a correction. Look at revised manuscript.

Thank you again to all reviewers for their valuable comments, suggestions, and interest in our work. We hope that we try to answer all your comments in the best way possible. Please let us know if there are other comments that we have not addressed because we are ready to learn from you.

With regards!

Thank you!

Yours sincerely

Correspondence authors
